# Prevalence of elephantiasis, an overlooked disease in Southern Africa: a comprehensive review

**DOI:** 10.1590/1678-9199-JVATITD-2024-0007

**Published:** 2024-10-14

**Authors:** Siphamandla Qhubekani Lamula, Elizabeth Bosede Aladejana, Emmanuel Adebowale Aladejana, Lisa Valencia Buwa-Komoreng

**Affiliations:** 1 Center of Infectious Diseases and Medicinal Plants, Department of Botany, Faculty of Science and Agriculture, University of Fort Hare, Alice, South Africa.; 2 SAMRC Microbial Water Quality Monitoring Center, University of Fort Hare, Alice, South Africa.; 3 Electron Microscopy Unit, Faculty of Science and Agriculture, University of Fort Hare, Alice, South Africa .; 4 Department of Biochemistry and Microbiology, Faculty of Science and Agriculture, University of Fort Hare, Alice, South Africa.

**Keywords:** Elephantiasis, Lymphatic filariasis, Wuchereria bancrofti, Southern Africa, Southern African Development Community (SADC), Prevalence, Sub-Saharan Africa, Mass drug administration (MDA)

## Abstract

Elephantiasis, also known as lymphatic filariasis (LF), is a debilitating condition characterized by the thickening of the skin and muscles, primarily affecting the limbs, genitalia, and female breasts. Lymphatic filariasis is a major global health concern, affecting approximately 120 million people worldwide and having a significant impact on people's quality of life, mobility, and socio-economic status. Although LF is endemic in many parts of the world, including Africa, it is a neglected issue in Southern Africa, with little information available. According to the World Health Organisation, approximately 882.5 million people in 44 countries worldwide are at risk of contracting LF, making it the second most common vector-borne disease after malaria. The primary goal of this review was to assess the prevalence of elephantiasis in the Southern African Development Community (SADC) region. Lymphatic filariasis is endemic in four of the sixteen SADC countries, three countries have administered MDA to the population that required it and they are now under post-intervention surveillance, while LF is no longer a public health problem in Malawi. Global efforts to eliminate LF have been hampered by the non-availability of MDA in some SADC countries such as Angola, Mozambique, Zambia, and Zimbabwe. Despite the implementation of mass drug administration programs, a review of the literature reveals gaps in knowledge about LF prevalence cases in SADC countries. Each country faces unique challenges and successes in combating LF due to varying levels of available data and healthcare infrastructure. Some SADC countries continue to bear the burden of LF-related diseases, necessitating ongoing disease prevention and elimination efforts. This review emphasizes the importance of ongoing research, data collection, and novel policies to combat the spread of elephantiasis disease in the SADC region and beyond.

## Background

Elephantiasis, commonly known as lymphatic filariasis (LF), is a set of conditions characterized by the thickening of the skin and underlying tissues, particularly affecting areas such as the limbs, male genitalia, and female breasts [1]. Lymphatic filariasis results from infection by parasitic filarial nematode worms belonging to the Filarioidea superfamily, primarily *Wuchereria bancrofti* which accounts for 90% of cases globally as well as *Brugia malayi* and *Brugia timori* [2]. Lymphatic filariasis is transmitted via mosquito vectors and leads to lymphedema and hydrocele with accompanying acute attacks [3]. Elephantiasis is divided into two types: lymphatic filariasis, caused by filarial worms, and non-filarial elephantiasis, which is frequently associated with irritating soil particles found in volcanic soils. Non-filarial elephantiasis can also be caused by different illnesses such as tuberculosis, leprosy, leishmaniasis, recurrent streptococcal infections, and sexually transmitted infections like lymphogranuloma venereum [3].

**Figure 1.**
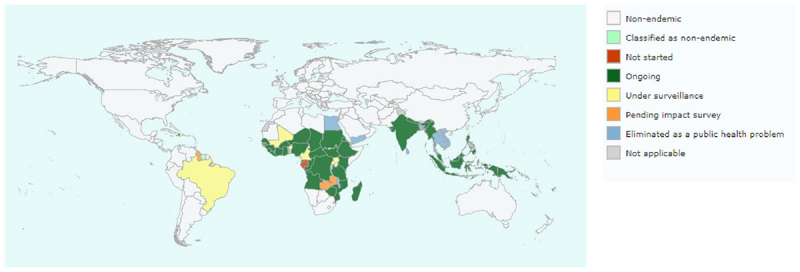
Map showing countries affected by lymphatic filariasis and the status of mass drug distribution [5].

The World Health Organization (WHO) estimates that 882.5 million people across 44 countries are residing in areas that require preventive chemotherapy (PC) to stop the spread of LF infection. Approximately 120 million individuals are affected (tropics and sub-tropics of Asia, Africa, the Western Pacific, and parts of the Caribbean and South America) as shown in Figure 1, with 40 million displaying chronic symptoms. Additionally, over 25 million men suffer from genital disease related to LF, and more than 15 million individuals are afflicted with lymphedema [4,5].

**Figure 2.**
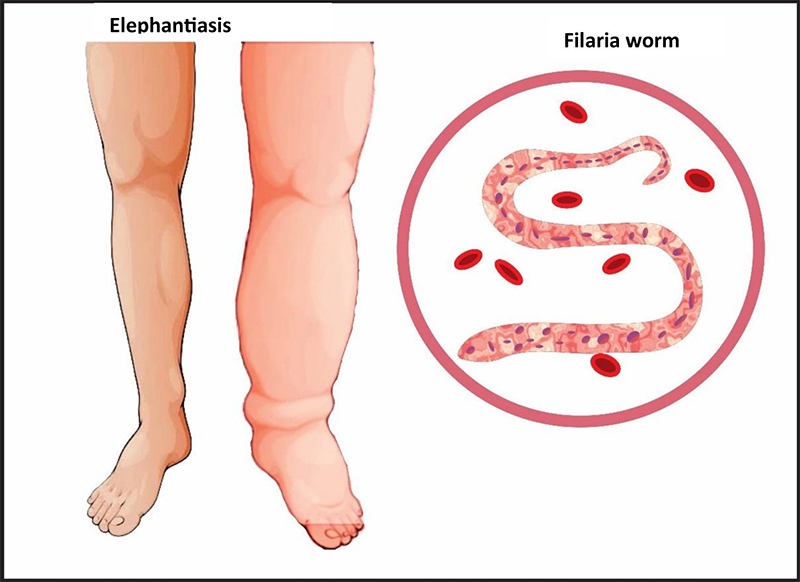
Leg of a person with elephantiasis.

As the second most common vector-borne disease after malaria, it poses a major global health burden [6-8]. Lymphatic filariasis, although not typically fatal, exerts a substantial toll through the pronounced impairment it inflicts. Swollen extremities and recurrent adenolymphangitis attacks (Figure 2) lead to significant suffering in affected individuals [9,10]. Beyond physical impairment, elephantiasis inflicts mental, social, and financial hardship through disability, stigma, lost work opportunities, and poverty [10]. 

Despite the global significance of LF, Southern African countries face neglect, and available data is notably sparse. Therefore, the primary objective of this study was to assess the prevalence of elephantiasis in the Southern Africa Development Community including Angola, Botswana, Comoros, Democratic Republic of Congo, Eswatini, Zimbabwe, Mozambique, Tanzania, Zambia, Malawi, Lesotho, Madagascar, Namibia, Seychelles, Swaziland, and South Africa, aiming to address a crucial gap in understanding LF's true extent in the region.

Global LF cases predominantly occur in India and Africa, with the remaining scattered across Southeast Asia, the Pacific, and the Americas [8]. In Africa, 39 countries are endemic with nearly 390 million at risk of infection [8,11]. Southern Africa is burdened not only by LF but also by a consortium of chronic infectious diseases, including schistosomiasis (primarily instigated by *Schistosoma haematobium, Schistosoma mansoni,* and *Schistosoma japonicum*), soil-transmitted helminthiasis (predominantly caused by *Ascaris lumbricoides, Necator americanus,* and *Trichuris trichiura*), blinding trachoma (attributed to *Chlamydia trachomatis*), leprosy (caused by *Mycobacterium leprae*), and Human African trypanosomiasis (caused by *Trypanosoma brucei*). This collective group of diseases impacts over 1.7 billion individuals globally, with a particular stronghold in tropical and subtropical regions [12]. 

*W. bancrofti* transmission involves 70 mosquito species globally, with *Anopheles gambiae*, *An. funestus, Culex pipiens*, and *Cx. fatigans* as primary vectors [13,14]*.* The primary LF vector in Africa is the *An. gambiae* complex [1,15]. 

In the Southern African region [Southern African Development Community (SADC)], lymphatic filariasis is endemic and poses a significant public health challenge in the region [16]. *Anopheles gambiae* has been identified as the primary vector in the region, with *Cx. quinquefasciatus* species playing a significant role [17-19]. *Culex quinquefasciatus* is commonly found in urban and semi-urban areas, while the *An. gambiae* complex and *An. funestus* group are prevalent in rural settings [20]. Sub-Saharan Africa accounts for 40% of the global LF burden, with reported cases ranging from 46 to 51 million in 2000 and 423 million at-risk individuals. In 2018, the total estimated at-risk population was 341 million, which required medical intervention [21]. The SADC region contributes over half of these LF infections, with Angola, Tanzania, Malawi, the Democratic Republic of Congo (DRC), Zambia, and Mozambique among the worst-hit countries [22-25]. While rural regions bear the most intense transmission, LF is also endemic in less developed peri-urban and urban areas [26]. 

Though the 2000-launched Global Program to Eliminate LF (GPELF) set 2020 targets, most Southern African nations lag in meeting elimination goals. The GPELF aimed to eliminate LF globally by 2020 through mass drug administration and control of morbidity [27]. The World Health Organization (WHO) Regional Office for Africa (AFRO) launched the Expanded Special Project for Elimination of Neglected Tropical Diseases (ESPEN) in 2016 intending to eliminate the five Neglected Tropical Diseases amenable to Preventive Chemotherapy (PC-NTDs) namely Onchocerciasis, Lymphatic Filariasis, Schistosomiasis, Soil Transmitted Helminthes and Trachoma, which collectively affect hundreds of millions of people in the continent. The program focuses on interrupting transmission and providing care for those already infected, and so far, it has recorded tremendous progress [5,28]. 

**Figure 3.**
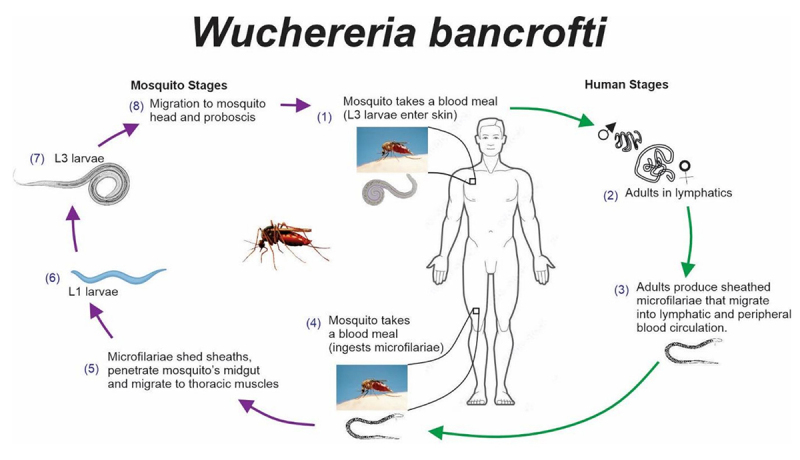
Etiology of lymphatic filariasis.

Despite comprehensive studies in some SADC countries, knowledge gaps persist, especially in Central and Southern Africa. Recent revelations challenge the historical belief of LF absence in Zimbabwe, emphasizing the need for updated research [6]. Beyond environmental factors, disease transmission (Figure 3) hinges on vector capabilities and the effectiveness of control initiatives. Insufficient disease surveillance contributes to this knowledge gap across the region. Thus, this study aims to review existing literature on elephantiasis prevalence in SADC countries, offering insights into LF epidemiology, disease burden, and consequences in the region.

### Search strategy and study selection

**Figure 4.**
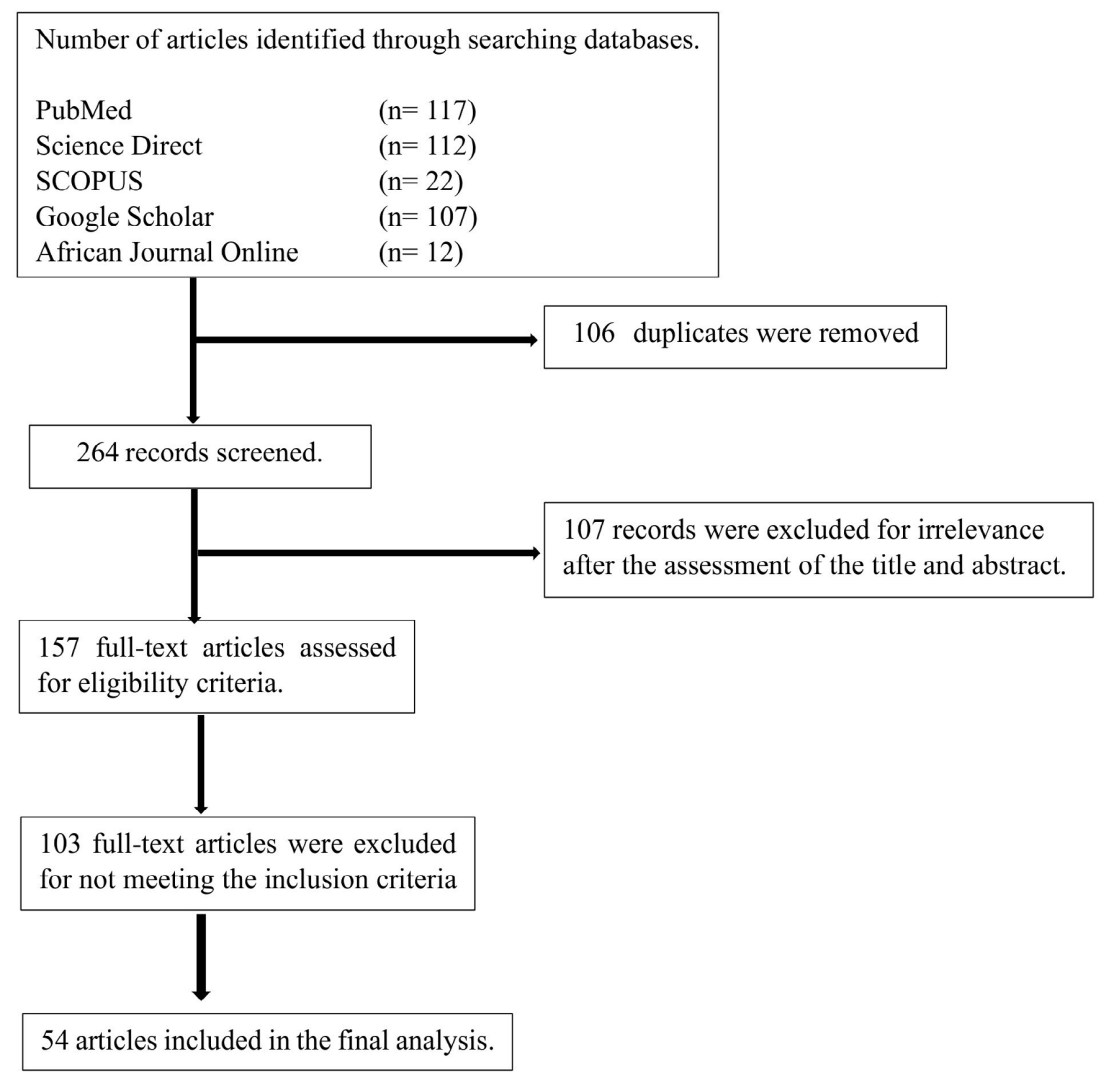
Flow diagram showing a selection process of eligible studies.

A comprehensive search of literature published in English on lymphatic filariasis between 2000 and 2023 was meticulously carried out and elucidated in Figure 4. This process involved the use of a variety of search engines, including PubMed, Science Direct, Google Scholar, Scopus, and African Journal Online (AJO), all of which served as search databases for data collection and analysis. The following set of keywords was used: {Health condition} Elephantiasis OR “lymphatic filariasis” OR lymphedema OR “filarial elephantiasis” OR “W*uchereria bancrofti*” OR “*Brugia malayi”* OR “*Brugia timori*” AND Prevalence OR rate OR Occurrence AND **{Southern African Country}** Angola OR DRC OR Botswana OR Lesotho OR Malawi OR Mozambique OR Namibia OR Tanzania OR South Africa OR Swaziland OR Zambia OR Zimbabwe AND Human. The keywords were carefully selected to aid in the precise retrieval and evaluation of relevant articles. The search returned 117, 112, 107, 21, and 15 articles from PubMed, Science Direct, Google Scholar, Scopus, and African Journal Online (AJO), respectively.

### Inclusion and exclusion criteria

The search was confined to peer-reviewed publications in the English language, with each article subject to rigorous evaluation against predetermined eligibility criteria. These criteria included the following: (a) original research articles originating from the Southern African Development Community (SADC) region, (b) epidemiological studies, clinical studies, vector studies, case reports and case series, surveillance and monitoring reports, intervention studies, genetic and molecular studies, policy and public health reports, and qualitative studies involving human subjects, (c) availability of the complete full-text document, and (d) a clear indication of the study's geographical location. Notably, papers presenting primary data on the prevalence, economic burden, costs associated with interventions, or implications for health systems in the context of control and elimination programs were selected for a more in-depth review. Additionally, papers featuring primary data on the costs related to any facet of treatment, prevention, or control were also included. On the other hand, the exclusion criteria encompassed, duplicates, unavailable full texts, or abstract-only papers, research done on non-Southern African communities, review articles, letters, comments to the editor, and animal studies. 

### Study characteristics and data extraction

Upon identifying studies that met the inclusion criteria, a comprehensive record was established, and a dedicated spreadsheet was generated to systematically organize and collate the pertinent information derived from these research articles. The spreadsheet meticulously cataloged the following details: author names, study titles, publication year, country of origin, gender, socio-economic classification (comprising low, middle, or high), residential status (urban or rural), educational background, population size under study, research findings, and study outcomes. The evaluation of the data's relevance predominantly relied on an initial assessment of the abstracts, followed by a thorough examination of the full-text articles. This process was initiated with an initial screening of titles and abstracts to gauge their conformity with the eligibility and relevance criteria established for this review, following the pre-defined inclusion and exclusion criteria. During this review, any books scrutinized were limited to those containing independently published papers that had subsequently been consolidated as book chapters. Additionally, to maintain the integrity of the review, duplicate studies were meticulously identified and subsequently expunged from the pool of selected research articles. The chosen papers were retrieved, read for data extraction, and subjected to a comprehensive review of the full-text content. The extracted data were diligently recorded in a structured tabular format for systematic analysis.

### Publication bias and limitations 

This review analyzed the existing literature reporting the prevalence of elephantiasis in the Southern African Development Community (SADC) countries, utilizing the specified inclusion criteria outlined above. The review focused on original research articles that provided data on study authorship, title, publication year, country, participant demographics (gender, socioeconomic status, residential status [urban or rural], educational background), population size under study, prevalence rates, research findings, and study outcomes. Articles were included if they reported extractable data on lymphatic filariasis prevalence in a SADC nation. Any article missing one or more pieces of information deemed necessary for quality appraisal was judged as having insufficient reporting or an elevated risk of bias, depending on the type of missing information. It is worth noting that the available data on the prevalence of elephantiasis in the SADC countries is notably scarce, contributing to the considerable challenge encountered during the compilation of this review.

### Epidemiological landscape of lymphatic filariasis in the SADC region

**Figure 5.**
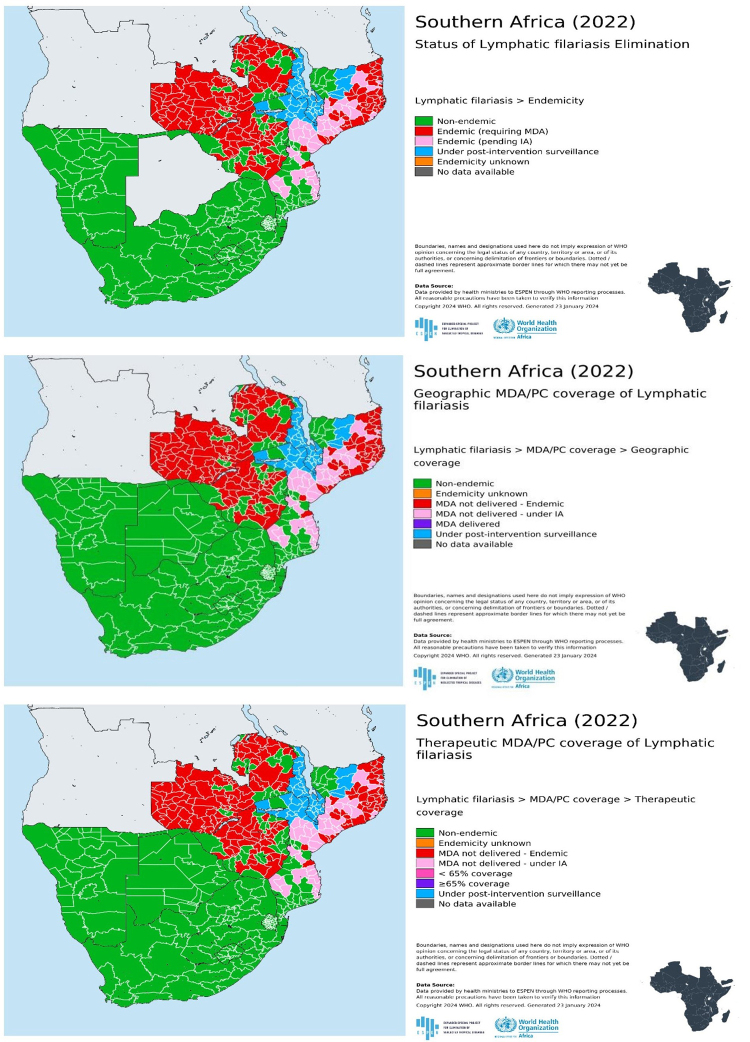
Endemicity, geographic MDA/PC coverage, and therapeutic MDA/PC coverage in Southern Africa [29].

The systematic search yielded a total of 370 articles from five databases. After screening the articles using the inclusion and exclusion criteria, a total of 54 articles published between the years 2000 and 2023 were deemed eligible for inclusion and are discussed in this review. All the eligible studies included in this review were from the Southern African Development Community region (SADC region) and the characteristics of all the eligible studies are summarized in Table 1. Different reports have shown that countries such as South Africa, Botswana, Lesotho, Namibia, and Swaziland are non-endemic regions and do not require preventive chemotherapy [5,29]. The Expanded Special Project for Elimination of Neglected Tropical Diseases (ESPEN) provided the 2022 Southern Africa regional map for the endemicity, geographic MDA/PC coverage, and therapeutic MDA/PC coverage of Lymphatic filariasis (Figure 5) [29].

**Table 1.** Characteristics of all the eligible studies conducted in SADC countries on lymphatic filariasis.

S/N	Country	Title	Age group	Gender	Socio-economic class (Low, middle, or high)	Place of residence (Urban or rural)	Educational status	Population size	Summary of the findings of the study	Reference
1.	Angola	Clinical, serological, and DNA testing in Bengo Province, Angola further reveals low filarial endemicity and opportunities for disease elimination	≥ 15 years old	615 males (38.1%) and 1001 females (61.9%)	Middle-income communities	Rural - sub-urban areas	Average educated	1616 individuals	None of the 1616 individuals surveyed in this study tested positive for LF antigen or by real-time PCR. In 16 localities, however, 1.7% had lymphedema and/or hydrocoele.	[10]
		Rapid integrated clinical survey to determine prevalence and codistribution patterns of lymphatic filariasis and onchocerciasis in a *Loa loa* co-endemic area: The Angolan experience	≥ 15 years old	761 males (37.9%) and 1246 females (62.1%)	Low income	Rural communities	Less educated	1558 individuals	None tested positive for LF infections among participants, except 1,7% of individuals who had lymphedema.	[30]
2.	DRC	Risk factors for lymphatic filariasis in two villages of the Democratic Republic of the Congo	≥ 5 years	383 males (46.7%)	Low-income communities	Rural communities	Less educated	1290 participants	The risk factor increased with age. Prevalence was higher in older poor males (46,1%) than in females.	[17]
		Filarial Antigenemia and *Loa loa* Night Blood Microfilaremia in an Area Without Bancroftian Filariasis in the Democratic Republic of Congo	≥ 14 years old	Randomized	Low-income community	Rural communities	Less educated	2,724 subjects	Positive ICT tests indicated the presence of filarial antigenemia and mf in 28 of 30 villages.	[24]
		Lymphatic filariasis in the Democratic Republic of Congo; micro-stratification overlap mapping (MOM) as a prerequisite for control and surveillance.	≥ 2 years old	-	Middle-income communities	Urban population	Average to well-educated	1290 participants	The findings suggest that the prevalence of LF appeared to vary within regions and across the country	[31]
3.	Malawi	Lymphatic filariasis morbidity mapping: a comprehensive examination of lymphedema burden in Chikwawa district, Malawi	22-90 years old	70% (48/69) were female and 30% (21/69) male	Low-income communities	Rural communities	Little-Basic education	76 participants	42% of the participants had lymphedema, reporting double figures compared to the previous years during the official MDA reports.	[22]
		Lymphatic filariasis in Lower Shire, Southern Malawi	>15 years old	A total of 241 (138 males, 103 females) and 296 (139 males, 157 females) individuals were examined clinically and for mf in Nchacha 18 and Belo, respectively.	Low-income communities	Rural communities	Less educated	48 villages (24 in each district) and 2431 households	Overall, In the two villages, 181% and 222% of people tested positive for microfilariae, and 63% and 66% tested positive for CFA, indicating a high prevalence of LF in the Lower Shire area of Malawi	[32]
		Identification of the vectors of lymphatic filariasis in the Lower Shire Valley, southern Malawi.	> 15 years old	Randomized	Low-income communities	Rural communities	Less educated	54 houses	*Anopheles gambiae* s.str. had the highest infection rates with all filarial stages and infective L3 larvae, followed by *A. arabiensis* and *A. funestus.*	[33]
		The geographical distribution of lymphatic filariasis infection in Malawi	≥ 15 years old	Female excess (64%) among the study participants	Low-mid income communities	Semi-urban areas	Low- to average education	2913 individuals	Based on ICT results, 9.2% of people tested positive for circulating filarial antigen (CFA). Males tested positive at a higher rate than females (11.0% versus 8.2%). However, the survey was not mapped out.	[34]
		Sentinel surveillance of Lymphatic filariasis, Schistosomiasis, Soil-transmitted helminths and Malaria in rural Southern Malawi	≥ 5 years old	50/50	Low-income communities	Rural communities	Less educated	1, 903 individuals	LF prevalence rates were lower than expected in all sentinel sites. Surveys utilizing a quick diagnostic antigen test reported a higher frequency of 30% in Chikwawa compared to 2.1% elsewhere.	[35]
		Quantifying the physical and socio-economic burden of filarial lymphedema in Chikwawa District, Malawi	> 18 years old	Female (70%) and 21 were male (30%)	Low-high income communities	Rural communities	Less-average income communities	69 individuals	The 69 individuals' mean overall disability score was 13.9, with a range of 8 to 34. There was no significant difference by sex, disease stage, frequency of ADLA, or duration of ADLA.	[36]
		Quantifying filariasis and malaria control activities in relation to lymphatic filariasis elimination: a multiple intervention score map (MISM) for Malawi	-	Randomized	Low-mid income communities	Rural communities	Less-average education	54 communities surveyed	This study's findings illustrate that the LF intervention program has successfully reached a considerable part of the population with MDA, with MISM covering most regions.	[37]
		Randomized controlled clinical trials of increased dose and frequency of albendazole and ivermectin on *Wuchereria bancrofti* microfilarial clearance in Northern Malawi	18-55 years old	Randomized	Low-income communities	Rural communities	Less educated	1851 individuals	After 12 months, both the normal MDA treatment and the experimental arms had high levels of microfilarial clearance, with additional clearance in all arms at 24 months.	[38]
		Measuring the physical and economic impact of filarial lymphedema in Chikwawa district, Malawi: a case-control study	Mean age of 58 and 55 years old, respectively	8 (26%) pairs were male and 23 (74%) pairs were female	Low-mid income communities	Semi-urban areas	Low- to average education	31 cases	Results showed that, Of the 31 cases, 26% of males had lymphedema compared to 23.1% of females and the mean age of affected individuals was 58 and 55, respectively. The majority had lymphedema in one leg.	[39]
		Significant improvement in quality-of-life following surgery for hydrocoele caused by lymphatic filariasis in Malawi: A prospective cohort study	≥ 18 years old	326 males	Low-income communities	Rural-urban communities	Basic education	326 males participants	Following surgery, approximately half of the men reported some pain/discomfort (55.9%), swelling (8.6%), bleeding (3.3%), and infection (5.9%), the majority of which had disappeared by 3-months, when most substantial improvements in their quality of life were discovered.	[40]
		Lymphatic filariasis in the Karonga district of northern Malawi: a prevalence survey	> 25% of adults	685 adult males; 769 adult females	Low income	Rural communities	Less educated	1,454 individuals	Results reveal a wider spread of *W. bancrofti* infections than previously reported.	[41]
		Elimination of lymphatic filariasis as a public health problem in Malawi	Children aged 6-7 years; adults > 40 years	Randomized	Low socio-economic class	Urban and rural areas across the 26 endemic districts nationwide	Mixed	Residents of 54 communities	Malawi successfully eliminated lymphatic filariasis (LF) as a public health problem in 2020 through the nationwide implementation of WHO's mass drug administration and morbidity management strategies. Challenges included the need for enhanced surveillance in low prevalence areas and addressing the large clinical burden, especially hydrocoele cases requiring surgery. 8,856 clinical LF cases were identified through extensive mapping, with most cases in three highly endemic districts. Post-elimination, sustained funding and partnerships are needed for surveillance, and patient care integration into health systems. The experience provides a model for other countries, and more research is recommended, especially in the post-elimination phase.	[42]
		The national distribution of lymphatic filariasis cases in Malawi using patient mapping and geostatistical modeling	Male: hydrocoele: 50.5 years; lymphedema: 54.4 years; with both conditions: 58.8 years. Female: lymphedema: 50.5 years.	Male: hydrocoele: 71.5%; lymphedema: 9.6%; both conditions: 1.0%. Female: lymphedema: 17.9%	Low to high-income	Both urban and rural areas.	Mixed	29,794 individuals (with a 95% credible interval of 26,957 to 32,927).	Geostatistical modeling estimated a total of 29,794 LF clinical cases, with 70.3% of cases in unmapped areas and 29.7% in mapped areas. The highest burden of LF was found in Chikwawa and Nsanje districts in the Southern Region and Karonga district in the Northern Region. The study highlights the importance of using existing LF antigenemia prevalence and clinical case data along with modeling approaches for resource allocation and long-term health strategies.	[43]
4.	Mozambique	Multiplex serology for impact evaluation of bed net distribution on burden of lymphatic filariasis and four species of human malaria in Nnorthern Mozambique	≥ 1 year old	Randomized	Low income	Semi-rural community	Average	1,320 individuals and 367 households per district	There was no statistically significant difference between the mosquito-causing malaria burden and the LF prevalence during the study.	[7]
5.	Tanzania	Safety and Tolerability of Ivermectin and Albendazole Mass Drug Administration in Lymphatic Filariasis Endemic Communities of Tanzania: A Cohort Event Monitoring Study	≥ 5 years old	Males and females	Low income	Rural communities	Less educated	10,000 individuals	The study reported treatment-associated adverse effects to be significantly higher among those who had pre-existing clinical conditions than those without.	[2]
		Management of Patients with Lymphedema Caused by Filariasis in Northeastern Tanzania: Alternative approaches	≥ 18 years old	-	Low income	Rural community	Less educated	46 (with 59 lymphedematous legs)	There was no statistically significant difference between those treated at the hospital and those receiving alternative treatment support.	[9]
		Change in composition of the *Anopheles gambiae* complex and its possible implications for the transmission of malaria and lymphatic filariasis in north-eastern Tanzania	-	-	Self-employment	Rural communities	Less educated	-	*Anopheles gambiae* complex had changed from being abundant to being rare, whilst *An. arabiensis* has changed from being the most rare to being the most abundant, which will have substantial implications for the epidemiology and control of malaria and lymphatic filariasis in the study area.	[15]
		Urban lymphatic filariasis in the metropolis of Dar es Salaam, Tanzania	≥ 5 years old	-	Low-income communities	Semi-rural periphery	Basic education	3655 community members	Despite haphazard urbanization, the study found a significant reduction in the burden of LF infection.	[16]
		Lymphatic filariasis control in Tanzania: infection, disease perceptions, and drug uptake patterns in an endemic community after multiple rounds of mass drug administration.	10-95 years old	Male to female ratio was 0.8	Low-income communities	Rural communities	Little to no education	1072 individuals	Poor people were the most affected. There was no statistically significant difference in drug uptake between those affected and who view LF infections as a health problem with those affected, but do not view LF as a health problem.	[18]
		Lymphatic filariasis elimination status: *Wuchereria bancrofti* infections in human populations and factors contributing to continued transmission after seven rounds of mass drug administration in Masasi District, Tanzania.	≥ 15 years old	63.6% (375/590) were female.	Low income	Rural community	Less educated	590 participants	The study reported a statistically significant decline in the prevalence of Wb infections. However, the prevalence of antigenemia remains above the World Health Organisation (WHO) recommendation threshold of 2%.	[19]
		Lymphatic filariasis transmission in Rufiji District, Southeastern Tanzania: infection status of the human population and mosquito vectors after twelve rounds of mass drug administration.	10-79 years old	The overall male-to-female ratio was 1.3	Low to middle-income	Rural community	Less educated	854 participants	Males were more infected than females, with 25 individual men having hydrocele. Infections were much higher in those who had not previously participated in any drug administration programs.	[20]
		Prevalence and Correlates of Lymphatic Filariasis Infection and Its Morbidity Following Mass Ivermectin and Albendazole Administration in Mkinga District, Northeastern Tanzania	≥ 14 years old	2045 males (49.7%)	Low income	Rural communities	Less educated	4115 individuals	The results revealed reductions in mf infections from individuals who followed the MDA programs.	[21]
		Lymphatic filariasis control in Tanga Region, Tanzania: status after eight rounds of mass drug administration	≥ 1 year old	Randomized	Low income	Rural community	Less educated	400 individuals	The study reported a statistically significant decrease in mf infections after eight rounds of MDA.	[23]
		Factors Influencing Drug Uptake during Mass Drug Administration for Control of Lymphatic Filariasis in Rural and Urban Tanzania.	≥ 15 years old	Females (64.2%); males (35.8%)	Low-to-middle-income communities	Rural and urban communities	Basic to average education	4053 community members	Individual views and practices in the target population were less important than easily modifiable provider-related factors in drug uptake.	[26]
		Acute adenolymphangitis due to bancroftian filariasis in Rufiji district, southeast Tanzania	≥ 10 years old	Males 1234; Females 1766	Low to middle-income	Rural community	Mixed	4576 individuals	Chronic filariasis was found in 3.1% (141) of the population. Individuals with lymphedema were more likely to have ADL attacks than the general population. ADL incidents were more common in people over the age of 40.	[44]
		Urban lymphatic filariasis in the city of Tanga, Tanzania, after seven rounds of mass drug administration	≥ 10 years old	Girl to boy ratio 0.9	Middle-income communities	Urban arias	Average to well-educated	1246 participants	The findings suggested a small decrease in infections among urban students. In contrast, no significant differences were observed in students from rural locations. Thus, the study corroborated the tendency of an inverse association between LF prevalence and transmission and socioeconomic levels, which has been shown in numerous studies in both urban and rural settings.	[45]
		Lymphatic Filariasis Control in Tanzania: Effect of Repeated Mass Drug Administration with Ivermectin and Albendazole on Infection and Transmission	≥ 1 year old	Male to female ratio was 0.96	Low income	Rural community	Less educated	1,112 individuals	The study reported a statistically significant decrease in microfilaria (mf) infections after MDA.	[46]
		Evidence of continued transmission of *Wuchereria bancrofti* and associated factors despite nine rounds of ivermectin and albendazole mass drug administration in Rufiji district, Tanzania.	6-9 years old	35.2% males; 64.8% females	Low-income communities	Rural communities	Less educated	413 children	Despite nine rounds of MDA, the findings show that LF transmission has persisted in the Rufiji district.	[47]
		Prevalence of Lymphatic Filariasis and Treatment Effectiveness of Albendazole/ Ivermectin in Individuals with HIV Coinfection in Southwest-Tanzania.	0-9 years old	51% females	Low-mid income communities	Semi-urban areas 3	Low- to average education	2104 individuals	LF prevalence was higher in males (26%) than in females (23.1%). In a high-prevalence location for both diseases, no difference in the initial prevalence of lymphatic filariasis was detected between HIV-infected and uninfected persons.	[48]
		Applying a mobile survey tool for assessing lymphatic filariasis morbidity in Mtwara Municipal Council of Tanzania	≥ 18 years old	37.0% of those opted-in, 384 male and 108 female) people completed the survey	Low-income community	Semi-urban community	Average educated	8,759 participants	For lymphedema and hydrocele, the proportion of patients reporting identical symptoms among friends and family was 66.0% and 70.9%, respectively. The data indicated that mobile phone-based surveys are a feasible method of conducting morbidity surveys.	[49]
		Lymphatic filariasis, infection status in *Culex quinquefasciatus* and *Anopheles* species after six rounds of mass drug administration in Masasi District, Tanzania.	-	-	Low income	Rural communities	Less educated	247 993 community members	After twelve rounds of MDA, the infection rate in this district was four times greater than the previously reported infection rate of 0.1% in Rufji district.	[50]
		Prevalence and management of filarial lymphedema and its associated factors in Lindi district, Tanzania: A community-based cross-sectional study.	≥ 18 years old	(56%) females,	Low income	Rural community	Primary education	954 community members	Nearly 50% of the participants had lymphedema and individuals above 50 years old were the most affected.	[51]
		Lymphatic filariasis patient identification in a Large Urban Area of Tanzania: An application of a community-led health system	≥ 15 years old	Prevalence per 100,000 total population (per 100,000 males and females)	High-income communities	Urban communities	Well educated	6889 patients	The prevalence of LF cases indicated a much higher the burden of LE and hydrocoele in Dar es Salaam than anticipated for an urban center, with 2251 patients reported to have LF, 4169 patients reported to have hydrocoele plus a further 469 patients having both conditions. The prevalence of LF was approximately equal between males and females in all three districts	[52]
		Association between Mannose-Binding Lectin Polymorphisms and *Wuchereria bancrofti* Infection in two Communities in North-Eastern Tanzania	≥ 1-70 years old	51 males and females 53	Low income	Rural communities	Less educated	104 individuals	The findings showed that. Among the 82 individuals who were mf negative in 1975, 50 (61.0%) were CFA negative in 2006, whereas among the 22 individuals who were MF positive in 1975, 20 (90.9%) were CFA positive in 2006.	[53]
		Increased HIV Incidence in *Wuchereria bancrofti* Microfilaria Positive Individuals in Tanzania	14 to 90 years old	170 (48.6%) females	Low to middle income	Rural to semi-urban communities	Less to average educated	350 individuals	12 (3.4%) tested positive for Wb microfilaria chitinase, with 9/170 (5.3%) positive results for samples from female participants and 3/180 (1.7%) from male participants.	[54]
		Community Participation in the Mass Drug Administration and their Knowledge, Attitudes, and Practices on Management of Filarial Lymphedema in Lindi District, Tanzania: A Cross-Sectional Study	18 to 87 years old	Female (56%)	Low to high income	Semi-urban to urban communities	Average to highly educated	954 study participants	83.9% reported having participated in the previous MDA rounds with more than three-quarters of them (78.5%) participated in ≤ 5 rounds while 21.5% participated in ≥ 6 rounds since the launching of the LF elimination program.	[55]
		A step towards the elimination of *Wuchereria* *bancrofti* in Southwest Tanzania 10 years after mass drug administration with Albendazole and Ivermectin	14 to 65 years old	654 (50.3%) females	Low to middle income	Rural to semi-urban communities	Less to average educated	1299 participants	The results indicated a reduction in the prevalence of lymphatic filariasis from 35.1% in 2009 to 27.7% in 2019 after seven years of treatment among the 14-65-year-olds. An additional three years of treatment further demonstrated a 1.7% reduction in LF prevalence.	[56]
		A 22-year follow-up study on lymphatic filariasis in Tanzania: Analysis of immunological responsiveness in relation to long-term infection pattern	34-74 years old	Males (26)/females (45)	Low income	Rural communities	Less to average educated	71 individuals	The finding showed no significant differences in infections after 22 years with 61 (85.9%) of the study individuals having the same infection status in 1975 and 1996.	[57]
		Soil-transmitted helminths and scabies in Zanzibar, Tanzania following mass drug administration for lymphatic filariasis - a rapid assessment methodology to assess the impact	≥ 5 years	Randomized	High income	Urban communities	Highly educated	34 815 cases	The findings indicated a 90-98% decline in soil-transmitted helminths and a 68-98% decline in scabies infections	[58]
		Current Epidemiological Assessment of Bancroftian Filariasis in Tanga Region, Northeastern Tanzania	≥ 5 years old	65.1% (307) males; 34.9% (165) females.	Low to high income	Rural to urban communities	Less to highly educated	472 individuals	The results revealed reductions in mf infections from individuals who followed the MDA program. However, there is clear evidence of ongoing transmission despite the eight rounds of MDA	[59]
		Cross-sectional relationship between HIV, lymphatic filariasis and other parasitic infections in adults in coastal Northeastern Tanzania	18-70 years old	517 (57%) females	Low to high income	Rural to urban communities	Less to highly educated	907 individuals	The results showed no relationship between HIV, lymphatic filariasis and other parasitic infections	[60]
		Lymphatic filariasis elimination efforts in Rufiji, Southeastern Tanzania: decline in circulating filarial antigen prevalence in young school children after twelve rounds of mass drug administration and utilization of long-lasting insecticide-treated nets	6 and 9 years old	236 (57.1%) were female and 177 (42.9%)	Low income	Rural communities	Less educated	413 children	The results indicated a reduction in *W. bancrofti* CFA in young school Children after 12 rounds of MDA	[61]
6.	Zambia	Lymphatic Filariasis Elimination Status: *Wuchereria bancrofti* (Wb) Infections in Human Populations after Five Effective Rounds of Mass Drug Administration (MDA) in Zambia	≥ 2 years old	(59%) females and (41%) males.	Low to high-income communities	Rural to urban communities	Mixed	47,235 participants (148 sites)	The survey found that Wb prevalence was higher in females than in males and the infections increased with the increase in the age of participants. Furthermore, the program’s elimination status falls short of the target of 0.0 infections by 2%.	[1]
		Lymphatic filariasis in Luangwa District, South-East Zambia	≥ 15 years old	Females (51.4%); males (48.6%)	Poor to low-income communities	Rural neighboring communities	Less educated	The study communities had 205 registered households	The prevalence of LF was slightly higher in males than in females and the infections increased with the increase in age.	[6]
		Health beliefs and health-seeking behavior towards lymphatic filariasis morbidity management and disability prevention services in Luangwa District, Zambia: Community and provider perspectives	18-50	50/50	Low income	Rural communities	Less educated	237 individual cases	The disease was well-known by the community members. The signs and symptoms of LF infection were well described by the community, except for the cause of it. The majority believe that it was caused by animal feces and witchcraft and traditional healers were more equipped to treat the infection than medical doctors.	[8]
		How community engagement strategies shape participation in mass drug administration programs for lymphatic filariasis: The case of Luangwa District, Zambia	≥ 18 years old	-	Low income	Rural community	Less educated	69 participants	The study noted that participation is greater from already existing structural groupings and the church commanded the largest group willing to participate when called through religious leaders.	[25]
		Significant decline in lymphatic filariasis associated with nationwide scale-up of insecticide-treated nets in Zambia	≥ 15 years old	Randomized	Low to high-income communities	Rural to urban communities	Less to high education levels	10,995 individuals	This study highlighted a significant decrease in LF prevalence across the country as well as the consistent growth in mass drug administration coverage.	[27]
		Mapping the Geographical Distribution of Lymphatic Filariasis in Zambia	12-96 Years old	6376 females; 3585 males	Low-high income communities	Rural-urban communities	Less-high educated	10193 volunteers	Positive CFA cases were found at 84 (77.8%) of the surveyed locations, with prevalence ranging from 1.0 to 53.9%. The frequency was 5% in 49 sites and 15% in 14.	[62]
7.	Zimbabwe	Multiplex peptide microarray profiling of antibody reactivity against neglected tropical diseases derived B-cell epitopes for serodiagnosis in Zimbabwe	≥ 11 years old	49.1% males	Low income	Rural community	Less educated	170 participants	Peptide microarray technology showed positive results for Wb*,* indicating the presence of a parasite thought not to exist in Zimbabwe.	[12]
		Spotlight on lymphatic filariasis and trachoma in Zimbabwe: Assessing baseline data for control program development.	≥ 14 years old	Randomized	Low-income communities	Rural communities	Less-basic education	Six hundred and fifty (650) participants	The study findings indicated that LF and trachoma are still poorly understood in Zimbabwe. The inclusion of vernacular words for the diseases' symptoms, on the other hand, suggests the presence of LF and trachoma in these places.	[63]

### Prevalence of elephantiasis in Angola

It is noteworthy that the Republic of Angola holds a prominent position as a priority nation for the elimination of onchocerciasis and LF within the sub-Saharan African context. In the pursuit of this goal, the country has adopted a proactive approach by implementing mass drug administration. This MDA strategy involves the utilization of a combination of ivermectin, albendazole, and diethylcarbamazine in various regimen combinations, all aimed at effectively interrupting the transmission of these diseases [64].

This review highlights a noticeable dearth of information concerning the incidence of elephantiasis cases in Angola. This observation raises the possibility that such cases either go unreported, lack proper documentation, or that the vectors responsible for causing elephantiasis are not prevalent in the region. In 2020, Paulo et al. [10] conducted a comprehensive research study, aiming to assess the prevalence of *Loa loa, Onchocerca volvulus*, and *W. bancrofti* infections within an under-researched region of Bengo Province, Angola. This extensive investigation involved the examination of 22 distinct communities and employed a multifaceted approach, combining clinical assessments, serological analyses, and DNA diagnostics to ascertain the prevalence of these infections. The study encompassed a total of 1,616 individuals (38.1% male, 61.9% female), with an average age of 43 years. The findings revealed that 6.2% of individuals had eyeworms, as determined by the rapid assessment procedure for loiasis surveys, and 11.5% by nested PCR analyses of venous blood. In addition, 4.7% of individuals tested positive for *O. volvulus* using the Ov16 ELISA method. Interestingly, none of the individuals were found to be positive for *W. bancrofti* infection, determined through antigen-based immunochromatographic tests and real-time PCR analysis. However, the study identified 27 individuals (1.7%) who were presented with clinical conditions associated with lymphatic filariasis (LF). Among them, there were 11 cases of lymphedema (0.68%), comprising six male and five female cases, 14 cases of hydrocoele in men (0.87%), and two men who exhibited both conditions (0.12%). The relatively low incidence of lymphedema and hydrocoele cases in the surveyed regions of Angola suggests that the burden may not be as extensive as in some other areas with a higher prevalence. In addition, Brito et al. [30] conducted a rapid integrated filarial mapping survey within Bengo Province, which is in the northern region of Angola. This survey was primarily based on the identification of readily observable clinical manifestations associated with lymphatic filariasis and onchocerciasis, a pragmatic approach that allowed for the timely determination of prevalence and co-distribution patterns within a resource-constrained setting. The study included 2,007 people from 29 different localities spread throughout five provincial municipalities. The rapid assessment method for loiasis (RAPLOA) and the rapid epidemiological mapping of onchocerciasis (REMO) were used to estimate community prevalence. In addition to these methods, two other studies were conducted on clinical manifestations of lymphatic filariasis (particularly, the existence of lymphedema and hydrocoele). The study found that out of the 29 communities surveyed, seven reported lymphedema cases, 12 reported hydrocoele cases, and three reported both clinical conditions, but different individuals were affected. While clinical cases are not always reliable indicators of recent transmission or endemicity, these comparatively low numbers correlate with previous surveys undertaken in the same region in the 1960s [65]. These findings highlight the possibility of isolated foci of *W. bancrofti* distribution in Northern Angola, with endemic zones detected in Cabinda District and the Northern section of Zaire District, as articulated by Kelly-Hope et al. [66]. The current prevalence of lymphatic filariasis in this region remains undetermined; however, it is anticipated to be at a low level. This projection is based on historical data and recent modeled map estimates as presented by Cano et al. [67] and the World Health Organization [29]. In 2020, over three million people required MDA as part of Angola’s MDA, 988,078 people received ivermectin plus albendazole, 86.8% of endemic regions were covered, and 47.3% of the individuals were treated as per the program target [68]. Due to the non-supply of MDA, the population of people who require MDA has increased to 4,105,407, hence, LF has been reported to be endemic in the country [69]. 

### Prevalence of elephantiasis in the Democratic Republic of Congo (DRC)

The World Health Organization [68] designated the DRC as the largest endemic country for LF in Africa, exposing over 49 million individuals to this health risk. However, the precise extent of this risk remains ambiguously defined, as recent comprehensive studies are conspicuously absent, and the available historical information is primarily derived from investigations conducted during the pre-independence era [70,71].

Kelly-Hope et al. [31] developed a novel methodology called microstratification overlap mapping in their study. This approach aims to identify regions that would be instrumental in the pursuit of LF elimination within the DRC. They underlined the scarcity of recent publications providing insights into LF's status in the DRC.

Another study by Bakajika et al. [24] mapped the Ituri and Haut Uele regions in the eastern part of the DRC, previously suspected to be LF-endemic areas before the initiation of MDA for LF elimination. Their study scrutinized individuals aged over 14 years, among which only one out of more than 2,700 tested was infected with *W. bancrofti.* The infection was identified through quantitative polymerase chain reaction (qPCR). Consequently, the authors concluded that MDA might not be necessary for these regions.

In contrast, Chesnais et al. [17] conducted an epidemiological study on LF in two endemic villages within the Republic of Congo. Their research found that 31.6% of individuals aged five years or older (out of 820 individuals) had *W. bancrofti* antigenemia, while 11.8% (97 out of 820) displayed microfilaremia. Multivariable analysis of risk factors for antigenemia revealed that males (adjusted odds ratio (aOR) = 1.75), older individuals (aOR = 9.12 for those aged over 35 years, 95%), non-users of bednets (aOR = 1.57), individuals engaged in farming (aOR = 2.21), and those residing near rivers (aOR = 2.78) faced an elevated risk. Hence, individuals who did not utilize bednets, engaged in agricultural activities, had not taken anthelmintics in the past year, and lived in proximity to the Nsitim River exhibited a substantially increased risk of circulating filarial antigen.

In 2022, over 45,243,848 people required MDA as part of DRC’s MDA, with over 36 million people treated with ivermectin plus albendazole, 100% of endemic regions were covered, and 97% of the individuals were treated as per the program target. The country is now declared to be under post-intervention surveillance [69].

### Prevalence of elephantiasis in Mozambique

Mozambique is considered one of the countries with the highest neglected tropical diseases (NTD) burden, and LF topping the five NTDs in the country [72]. According to Manhenje et al. [64], LF is endemic in Mozambique, where it is caused by *W. bancrofti* with *Cx. quinquefasciatus* as the main vector. Before 1953, no cases of LF were identified in Mozambique until the campaign of the Mission to Combat Trypanosomiasis. It was during this campaign that 14 cases (0.26% of prevalence) of *Wuchereria brancrofti* infection in blood in the human African trypanosomiasis endemic area were detected. From 1995 through 2000, hospital histories in Pemba (Cabo Delgado) showed 1300 cases identified, of which 99.5% of those had hydroceles and the only cases of lymphedema identified were in women. In addition, there was a lower frequency of cases in individuals below 15 years old [72]. 

In 1985, Fujita et al. [73] investigated the prevalence of microfilariae of bancroftian filaria in the region of Quelimane, situated within Mozambique, East Africa. The study revealed the detection of microfilariae in a male individual out of the eight Africans included in the examination, with the identified filarial species appearing to be *W. bancrofti*.

Manhenje et al. [64] conducted a comprehensive investigation into the socio-environmental determinants and transmission risk associated with LF in Central and Northern Mozambique. Their findings indicate a significant relationship between LF transmission and specific environmental factors. It was observed that LF transmission rates exhibited an upward trend with higher mean maximum temperatures, while a reverse correlation was noted with increasing altitude. The relative stability of annual temperatures, especially in tropical regions, coupled with altitude, prevailing economic conditions, and the dominant crop, which is rice cultivation, emerged as influential factors contributing to the presence and abundance of the LF vector. Intriguingly, even though the LF vector was present in the hinterland areas, it was not conducive to the presence and survival of the LF parasite. The transmission risk was notably elevated in Zambezia, consequently resulting in a higher prevalence of LF in that region, while Niassa exhibited an opposite scenario. The study's overarching conclusion underscores the significance of temperature, altitude, and socio-economic development status, particularly in urban areas, as essential factors to be considered in assessing the transmission risk of LF in Mozambique. These findings provide valuable insights into the multifaceted dynamics of LF transmission in this region.

Plucinski et al. [7] conducted a study to explore the utility of multiplex serology in assessing the impact of bed net distribution on the prevalence of lymphatic filariasis in Northern Mozambique. The emergence of multiplex antibody-detection technology has introduced a novel opportunity to concurrently evaluate the effects of control measures on the prevalence of several diseases. The research focused on an area where noteworthy reductions in LF seropositivity were observed. However, it is important to note that these reductions were not found to be associated with the usage of long-lasting insecticidal nets (LLINs). The study suggests that the MDA efforts could have potentially obscured any discernible impact of LLINs on the LF seropositivity within the population. In 2020, over 19 million people required MDA as part of Mozambique’s MDA, with over 14 million people treated with ivermectin plus albendazole, 98.9% of endemic regions were covered, and 76.9% of the individuals were treated as per the program target [68]. However, due to the non-supply of MDA, over 9 million people are yet to be treated, making LF endemic in the country [69].

### Prevalence of elephantiasis in Zimbabwe

Lymphatic Filariasis is one of the top four Neglected Tropical Diseases (NTDs) in Zimbabwe, alongside other NTDs such as bilharzia, intestinal worms, and blinding trachoma. Collaborative efforts between the Government of Zimbabwe, the Ministry of Health and Childcare, the Higher Life Foundation, and the World Health Organization (WHO) are currently underway to formulate a comprehensive strategy for mass treatment aimed at curtailing LF transmission [74].

Regrettably, the existing data on the potential endemicity of LF in Zimbabwe, as well as the knowledge, attitudes, and practices (KAP) of local communities regarding this disease, are scarce. A survey conducted by Vu et al. [63] revealed alarming findings. Among 650 participants aged above 14 years who partook in a KAP questionnaire, a staggering 95.5% were unable to identify specific signs of LF, and 99.5% remained ignorant of the disease's cause. However, when the symptoms of LF were explained in the local language, 42.2% were able to provide vernacular terms for 'hydrocele.' Notably, about 44.2% strongly concurred that LF is a prevailing issue within their community, and an overwhelming 91.1% expressed their willingness to participate in MDA as a means of LF control. In addition, a study by Vengesai et al. [12] profiling antibody reactivity against neglected tropical diseases has disclosed the presence of LF in 39 districts in Zimbabwe, emphasizing the scope of this health concern in the region. These findings underscore the prevailing limited understanding of LF in Zimbabwe. As of 2022, over eight million people in Zimbabwe required MDA, this is a result of the non-rollout of MDA, hence making LF endemic in the country [69]. 

### Prevalence of elephantiasis in Zambia

In the preceding year, there was a paucity of documented reports regarding LF in Zambia. It is important to note that LF is now prevalent in 96 out of the 116 districts within Zambia, as reported by Shirley et al. [75]. These endemic areas, inhabited by approximately 11 million individuals, face an increased risk of LF transmission, as highlighted by Chitalu [76]. Historically, the earliest instances of *W. bancrofti* microfilaremia in Zambia date back to 1938, according to research by Buckley in 1946 [77]. During his survey on human helminth infections in the northern and Central regions of the country, Buckley identified three cases. The author suggested that LF was possibly contracted elsewhere, as the afflicted individuals were either transient residents in Zambia or had visited LF-endemic neighboring countries. In 1976, Hira documented the first confirmed autochthonous case of *W. bancrofti* infection within Zambia. This case involved a 25-year-old fisherman from Luangwa District, who had never traveled abroad. The patient presented symptoms of a tender swelling in the right inguinal fossa and swollen ankles, as reported by Hira [78]. Subsequently, Hira continued to report additional cases of LF infection, including autochthonous cases, in his publications in 1975 and 1977 [79]. In addition, cases of LF infection emerged in Zambia, with two notables of a 22-year-old male and a 46-year-old female from different regions of the country, both of whom exhibited microfilariae in their blood smears. In the latter case, the female also presented symptoms of elephantiasis, which was suspected to be a consequence of the *W. bancrofti* infection [6].

In alignment with the World Health Assembly's (WHA) 1997 resolution to globally eliminate LF as a public health concern, the Zambian government embarked on an extensive LF mapping survey spanning from 2003 to 2011. Volunteers from all districts across the country underwent examinations for the presence of circulating filarial antigen, which serves as an indicator of *W. bancrofti* adult worm infection, as per the WHO guidelines [80]. The study determined a nationwide disease prevalence of 7.4%, with some regions, notably Western province, exhibiting considerably higher prevalence rates, reaching up to 53.9%. Subsequently, the government of Zambia has played a significant role in reducing LF cases, in line with global elimination objectives. This endeavor has been facilitated through the implementation of MDA programs and other public health campaigns, as emphasized by Chitalu [76].

Shawa et al. [6] evaluated the infection, disease transmission, and human perception aspects of LF in the endemic region of Luangwa District, located in Southeast Zambia. The study encompassed 985 individuals aged one year and older, focusing on infection rates, disease prevalence, transmission dynamics, and the community's perception of the disease. The findings from the research revealed a notable increase in the prevalence of circulating filarial antigens (CFA) with advancing age. Specifically, CFA prevalence ranged from 1.2% in the age group of 1 to 14 years to 20.6% in individuals above the age of 50, resulting in an overall prevalence of 8.6%. Furthermore, the study identified *W. bancrofti* microfilariae in 10.9% of individuals who tested positive for CFA, which corresponds to a community prevalence of 0.9%. The investigation also observed a substantial variance in the prevalence and intensity of Bm14 antibodies, a marker indicating exposure to LF transmission. Individuals aged 30 years and above exhibited significantly higher rates of Bm14 antibodies compared to their younger counterparts. While elephantiasis and hydrocele were recognized as well-known clinical manifestations in the area, the study population only presented a single case of hydrocele. Despite this, the study effectively confirmed the endemic status of LF within the study communities. However, the prevalence of LF infection and associated diseases within the population was found to be relatively low.

Furthermore, Mwase et al. [62] investigated the geographical distribution and prevalence patterns of LF in Zambia. The research encompassed the assessment of roughly 10,000 willing adult volunteers hailing from 108 precisely geo-referenced survey locations spanning Zambia. This assessment involved the detection of circulating filarial antigens (CFA) using rapid format Immunochromatographic Test (ICT) cards. Subsequently, the gathered data was utilized to generate a map illustrating the prevalence of CFA in different regions of Zambia. The research findings revealed that an impressive 78% of the surveyed locations exhibited cases of CFA positivity, with prevalence rates spanning a range between 1% and 54%. It is noteworthy that, although most of the positively identified survey locations showed relatively low prevalence rates, the study did identify six specific areas with a prevalence exceeding 15%.

Matapo et al. [1] conducted a post-Mass Drug Administration (MDA) pre-transmission assessment survey (pre-TAS) across 80 districts distributed throughout nine provinces within Zambia. The primary objective of the investigation was to ascertain whether the prevalence of LF had been successfully reduced to levels below 2% in terms of antigenemia and less than 1% in microfilaremia. The results of the survey revealed a notable outcome, with 79 out of the 80 endemic districts displaying a meager prevalence of *W. bancrofti* antigens at 0.14%, and a complete absence of microfilariae. It is important to emphasize that all districts, except for Chibombo district, achieved the desired objective of maintaining a prevalence below 2% for antigenemia. Furthermore, it was observed that most individuals testing positive for Wb Antigens had undergone two, three, or four rounds of Mass Drug Administration, while individuals who had only received a single round of MDA surprisingly did not exhibit circulating antigens of *W. bancrofti*.

The MDA initiative for the LF program in Zambia is administered by the NTDs Unit, which operates under the jurisdiction of the Department of Public Health within the Ministry of Health. In the year 2020, the program saw a significant demand, with more than 12 million individuals requiring a combination of diethylcarbamazine citrate (DEC) at a dosage of 6 mg/kg and Albendazole at 400 mg as part of Zambia's MDA efforts. The program effectively treated over six million individuals, covering 55.3% of the endemic regions, and achieving treatment for 89.2% of the targeted individuals, following the program's objectives as outlined by the World Health Organization [68]. However, due to the shortage of MDA, over 12 million people are now expected to be treated, making LF endemic in Zambia [69].

### Prevalence of elephantiasis in Malawi

Nielsen et al. [32] conducted the first epidemiological survey within the Lower Shire region, covering the Nsanje and Chikwawa Districts of Southern Malawi, with the primary objective of ascertaining the presence of LF. The survey encompassed a comprehensive approach, including the utilization of a structured questionnaire, the ICT whole-blood test to detect the *W. bancrofti-*specific circulating filarial antigen (CFA), and a rigorous parasitological examination that involved the assessment of CFA and clinical scrutiny. A total of 1,130 and 1,301 individuals actively participated in the Nsanje and Chikwawa Districts, respectively. The study was conducted in three phases, and in the two villages included in the third phase, which were selected based on high CFA prevalence, the latter was 62.3% and 64.6%, and the prevalence of microfilaremia was 18.1% and 22.2%, respectively. Furthermore, in the same villages, among individuals aged 15 years and older, 3.7% and 1.3% exhibited symptoms of leg elephantiasis, while 17.9% and 13.0% (limited to males) were afflicted with hydrocoele. Consequently, the study underscored the high endemicity of LF within the Lower Shire area of Malawi, emphasizing the urgency for proactive measures and interventions to control this disease.

Likewise, Ngwira et al. [41] conducted a comprehensive investigation into the prevalence of LF within the Northern Malawian district of Karonga. The study revealed that, among the 12 villages sampled, over a quarter of the adult population (> 25%) tested positive for *W. bancrofti* antigenemia based on the results of 687 immunochromatographic tests (ICTs). Village-specific prevalence rates ranged from 28% to 58%. Furthermore, when subjecting 685 adult male residents to full-body clinical examinations, approximately 11.7% were diagnosed with hydrocele, while lymphedema affected 1.0% of these adult males and 3.7% of the adult female residents subjected to examination. Microfilariae were identified in 30.8% of the 107 thick smears created from night-blood samples obtained from individuals who exhibited positive ICT test results.

Furthermore, Merelo-Lobo et al. [33] conducted an extensive examination of the vectors responsible for transmitting LF in Malawi. The research findings revealed the prevalence of filarial infection and the carriage of infective larvae in key mosquito species. Specifically, *Anopheles funestus*, *An. arabiensis,* and *An. gambiae sensu stricto* exhibited substantial rates of filarial infection, ranging from 2.2% to 3.1%. Of these vector species, *An. funestus* predominated, constituting 77.6% of the collected species and serving as the primary vector during the survey. This critical information sheds light on the dynamics of LF transmission in Malawi and underscores the significance of *An. funestus* in this context.

In addition, Ngwira et al. [34] carried out a comprehensive nationwide survey aimed at mapping the prevalence of LF in Malawi. This study encompassed 35 villages across 23 districts, and the findings unveiled a considerable variation in antigenemia prevalence as determined by ICTs, spanning from 0% to 35.9% in all the surveyed villages. The outcomes of this study strongly indicate that the regions adjoining the lake shore, the Phalombe Plain, and the lower Shire Valley should be accorded priority status within the framework of Malawi's LF elimination program.

The sentinel surveillance of LF as investigated by Msyamboza et al. [35] within the Chikwawa region of Southern Malawi, showed that the prevalence of microfilaremia was documented at 1.5%. Notably, this prevalence displayed minimal variation across the various sentinel sites, with rates ranging from 1.0% to 2.1%. Interestingly, the reported prevalence estimates of LF proved to be lower than anticipated, falling below the expected threshold of less than 2% across all the sentinel sites.

In 2014, Martindale et al. [36] and Smith et al. [22] investigated the occurrences of lymphedema within the purview of a single healthcare center catchment area situated in the Chikwawa district of Southern Malawi [22,36]. The study outcomes disclosed the existence of a total of 69 cases of lymphedema, representing an incidence rate of 32 cases per 10,000 population. Among these cases, 70% were female (48 cases) and 30% were male (21 cases). Of the identified cases, the majority, specifically 51 out of the 69 cases, exhibited symptoms falling within Dreyer stages 2-3, signifying advanced stages of the condition. Remarkably, nearly all the individuals, specifically 65 out of the 69 cases, had experienced acute episodes or attacks attributable to their lymphedema condition.

Some investigations conducted within the Southern Region's Chikwawa district have primarily centered on two key areas of research. First, the estimation of the precise number of individuals experiencing the clinical manifestations of LF [37]. Second, subsequent studies have sought to measure the self-assessed quality of life among individuals affected by LF [36,39]. These research endeavors collectively contribute to a deeper understanding of the impact of LF on affected populations and the broader implications for public health and well-being.

The national LF elimination program in Malawi has achieved significant success by effectively reducing disease transmission through the implementation of annual mass drug administration featuring ivermectin and albendazole [37,38,81]. Consequently, the program has transitioned to a phase where it can concentrate its efforts on diminishing the burden of associated morbidity, a strategic shift outlined by Stanton et al. [39]. The total population of people that required MDA has been treated, covering 100% of the endemic regions, and achieving treatment for 100% of the targeted individuals, hence, LF has been eliminated as a public health problem in Malawi [42,69].

### Prevalence of elephantiasis in Tanzania

Tanzania is one of the leading countries in sub-Saharan Africa burdened with a high prevalence of LF [18]. The National Lymphatic Filariasis Elimination Program (NLFEP) in Tanzania was initiated in 1997, prompted by a resolution passed at the World Health Assembly, which aimed to eliminate lymphatic filariasis as a public health concern by 2020 [82]. The Tanzania NLFEP embarked on its initial MDA campaign utilizing ivermectin and albendazole in the year 2000, treating 45,000 individuals. Over nine years, this program extended its coverage to encompass six regions and 34 districts, effectively treating a total of 9.2 million people. As of 2022, Tanzania had conducted 16 rounds of MDA [2]. Approximately 34 million individuals in Tanzania remain at risk of LF infection, with an estimated six million already affected [82]. The endemicity of LF in Tanzania varies significantly; it is highly endemic along the coastal regions, where antigenemia levels range from 45% to 60%, while areas in Western Tanzania exhibit lower endemicity levels, typically ranging between 2% and 4%. Furthermore, the regions between these extremes, such as Central Tanzania, the Southern Highlands, and North, and Northwestern Tanzania, exhibit varying degrees of endemicity.

In a study conducted by Bernhard et al. [9], the management of 46 patients with a total of 59 lymphedema legs attributed to filariasis in Northeastern Tanzania was documented. This research provided conclusive evidence of the presence of elephantiasis in the region. Simonsen et al. [46] report highlighted the impact of the NLFEP intervention on the prevalence of human microfilaremia. The study observed a rapid and statistically significant reduction in microfilaremia, with prevalence rates decreasing by 21.2% and 40.4%, and mean intensity decreasing by 48.4% and 73.7% after the first and second MDA cycles, respectively. Subsequently, the effectiveness of the intervention plateaued. The initial decline in microfilaremia also translated into substantial reductions in vector infection and infectivity rates, resulting in a considerable reduction in transmission, with decreases of 74.3% and 91.3% compared to pre-treatment levels after the first and second MDA rounds, respectively. However, it's noteworthy that the decrease in infection and infectivity rates eventually leveled off, and low-level transmission persisted after the third MDA. It is essential to emphasize that the MDAs had a limited impact on circulating filarial antigens and the antibody response to Bm14.

Simonsen et al. [23] in their study, delved into the assessment of LF control within the Tanga Region of Tanzania after the implementation of eight rounds of the MDA. The research revealed notable reductions in LF prevalence following the eighth MDA cycle. Specifically, the prevalence of CFA and microfilariae (mf) in communities decreased by 75.5% and 89.6% from baseline, reaching levels of 15.5% and 3.5%, respectively. Among schoolchildren, the prevalence of CFA dropped to 2.3%, marking a 90.9% reduction from baseline. Intriguingly, the prevalence of chronic LF morbidity after the eighth MDA round was less than half of the initial records. Moreover, no infective vector mosquitoes were detected after the seventh MDA. However, spot checks conducted in other districts after the eighth MDA indicated relatively high LF burdens in the coastal regions. 

Similarly, Jones et al. [47] scrutinized the status of lymphatic filariasis transmission after nine rounds of MDA administration in the Rufiji district, Tanzania. The study employed a cross-sectional survey and measured the presence of *W. bancrofti* CFA) in blood samples using immunochromatographic test cards. Among 413 standard-one schoolchildren tested, 14.3% tested positive for CFA. Notably, the prevalence of CFA was significantly lower in younger children (6.4%) compared to older children (40.4%). Hence, the authors concluded that LF transmission persisted in the district despite the implementation of nine rounds of MDA.

In a study conducted by Kroidl et al. [48], an examination of 2,104 individuals, ranging in age from 0 to 94 years, before receiving anti-filarial treatment in Southwest Tanzania, revealed an LF prevalence of 24.8%. Interestingly, LF was relatively rare in children, but its prevalence increased significantly in individuals aged 10 years and above. Among adults aged 18 years and older, the prevalence of LF was notably high at 42.6%. Furthermore, out of the initial 2,104 individuals, a subset of 798 were re-tested following two rounds of antifilarial treatment. The findings indicated a significant reduction in the prevalence of circulating filarial antigen from 21.6% to 19.7% after treatment, demonstrating a marked improvement. Similar findings were reported in a different study by Mnkai et al. [56], who investigated the prevalence of LF infection 10 years after the initiation of mass antifilarial medication administration among adults aged 14 to 65. The study's findings demonstrated that the prevalence of LF has decreased from 35% to 27.7% two years after the MDA was introduced. In the second analysis, the prevalence of LF dropped from 33.7% to 29.3%. These findings revealed a remarkable reduction in LF prevalence following the MDA interventions. In a different study, Mnkai et al. [54] investigated the link between increasing HIV occurrences in *Wuchereria bancrofti* microfilaria-positive persons in Tanzania. According to the study, 22 of 350 patients infected with *Wuchereria bancrofti* became HIV positive (1.98 per 100 PY). Using a multivariable analysis, the study found that the incidence of HIV was 4.58 times greater among Wb MF chitinase-positive persons than among Wb-infected but MF-negative individuals. The study's findings indicate that persons infected with *W. bancrofti* have a 2.3-fold greater HIV incidence than filarial-uninfected individuals. Similarly, in 2005, Nielsen et al. [60] investigated the cross-sectional relationship between HIV, lymphatic filariasis, and other parasitic infections in adults in coastal Northeastern Tanzania. Overall, the results were 7.9% for HIV, 43.5% for *Wuchereria bancrofti*-specific circulating filarial antigen (CFA), 12.3% for *P. falciparum*, 1.2% for *A. lumbricoides*, 7.1% for *T. trichiura*, and 75.7% for hookworm. The findings of the study showed a favorable connection between HIV infections and *W. bancrofti*-specific CFA and malaria, but an inverse association with hookworm infection and other parasites.

Furthermore, in a separate study by Mwingira et al. [83] the prevalence of lymphedema and hydrocele in the Mtwara Municipal Council was estimated using a mobile phone-based survey. The study engaged 492 survey participants (384 male and 108 female). It was observed that 20.9% of respondents reported signs of lymphedema, while 20.6% reported signs of hydrocele. Notably, a substantial proportion of hydrocele patients (59.5%) and lymphedema patients (46.6%) had actively sought treatment for their condition. Additionally, a significant number of patients reported similar symptoms among their friends and relatives, with rates of 66.0% for lymphedema and 70.9% for hydrocele. In another study of a health community-led door-to-door survey approach using the SMS reporting tool Measure-SMS-Morbidity in Dar es Salaam, Mwingira et al. [52] discovered that out of 6889 patients reported, 4169 had hydrocele, 2251 had lymphedema -elephantiasis, and 469 had both. Furthermore, LF patients with severe cases accounted for approximately a quarter (26.9%) of those reported, with the incidence of acute attacks increasing with reported LE severity. Similarly, Mshana et al. [59] assessed the status of Bancroftian filariasis infection rate and morbidity in the Tanga Region, Northeastern Tanzania, after MDA was disseminated and administered for over eight rounds. The study found that 5.51% of the 272 participants tested positive for CFA, with males accounting for the majority (3.3%). The study's findings revealed clear evidence of continued transmission despite eight rounds of MDA.

Jones et al. [61] examined *W. bancrofti* circulating filarial antigen (CFA) in young school pupils in the Rufiji district of Southeastern Tanzania after twelve rounds of MDA Mass. The results showed that 14.3% of the 413 children screened positive for CFA in 2012, compared to 0.0% of the 659 children tested in 2015. The study also found that the baseline *W. bancrofti* CFA in adults ranged from 49% to 64% before the implementation of MDA programs (unpublished data). Following the ninth and twelfth rounds of MDA, the prevalence of *W. bancrofti* CFA was observed to be around 14.3% and 0.0%. This showed a significant reduction in *W. bancrofti* CFA prevalence in young children. Similarly, in 2018, in a separate study, Jones et al. [20] assessed LF infection in the Rufiji district, located in southeastern Tanzania, following the implementation of twelve rounds of MDA Mass. The study involved 854 participants, and the findings revealed that 1.1% of them tested positive for CFA, with 0.1% found to be microfilaremic. Moreover, the prevalence of hydrocele and elephantiasis was determined to be 4.8% and 2.9%, respectively. Notably, the study identified a complete absence of *W. bancrofti* infection in any of the captured filarial vectors. Furthermore, none of the tested vectors using qPCR showed any signs of infectivity. These findings collectively indicated a substantial decline in the prevalence of LF and other transmission-related indices, suggesting that local transmission had become exceedingly rare or potentially non-existent within the study areas. Mohammed et al. [58] found similar results in their investigation of the impact of mass drug administration on lymphatic filariasis in Zanzibar, Tanzania. The findings revealed a considerable decrease in both soil-transmitted helminths and scabies infections, ranging from 90-98% and 68-98% drop, respectively. Following MDA, both soil-transmitted helminth and scabies infections decreased significantly. 

In 2020, a surveillance study conducted by Fimbo et al. [21] investigated the prevalence and associated factors of LF infection in the Mkinga district, located within the Tanga region of Tanzania. The study encompassed a total of 4,115 individuals, with 49.7% being male and 35.2% children. These individuals were screened for the presence of CFA, microfilaremia (mf), and manifestations of the disease across 15 villages. The findings of this study revealed an overall CFA-positivity prevalence of 5.8%, with notable variation observed between villages, ranging from 1.2% to 13.5%. CFA-positivity was more pronounced in males (8.8%) compared to females (3.3%), and it displayed a positive correlation with increasing age. Among those who tested CFA-positive, the prevalence of mf was recorded at 5.2%. Furthermore, it was observed that only 60% of eligible residents in the study area had undergone Drug Administration in the preceding year. Notably, CFA-positivity was twice as high among individuals who had missed MDA, which corroborates the findings of Lupenza et al. [50]. The study also examined the prevalence of specific LF-related manifestations. It indicated a prevalence of 6.4% for scrotal enlargement, 3.7% for hydrocele, 1.35% for arms or legs swelling, 1.2% for lymphedema, and 0.32% for lymphadenopathy. These acute and chronic pathologies impose significant barriers to socioeconomic development and contribute to an exceedingly poor quality of life, as highlighted by the World Health Organization [84].

Lupenza et al. [50] study focused on the evaluation of *W. bancrofti* infection in *Cx. quinquefasciatus* and *Anopheles* species after six rounds of MDA in the Masasi District of Southeastern Tanzania. The research encompassed an analysis of 365 pools of *Cx. quinquefasciatus* using PCR, which revealed that 33 of these pools were positive for *W. bancrofti*, equating to an infection rate of 0.5%. In contrast, all tested Anopheles species yielded negative results. *Wuchereria bancrofti* was not detected in 1,859 dissected mosquitoes analyzed through microscopy. Consequently, the study concluded that despite the six rounds of MDA, there was still evidence of low-level LF transmission persisting in the Masasi District.

In 2021, Lupenza et al. [19] conducted a follow-up investigation to assess the *W. bancrofti* infection status in individuals aged 15 years after seven rounds of MDA in the Masasi District, Southeastern Tanzania. Among the 590 participants, 30 individuals (5.1%) tested positive for CFA, and one individual (0.2%) was found to be positive for microfilaria of *W. bancrofti*. The study also revealed that compliance during the seventh round of MDA in 2019 was at 56%, falling below the minimum coverage recommended by WHO. This suboptimal compliance could potentially account for the observed research findings.

Mwakitalu et al. [16] investigated the urban lymphatic filariasis infection and transmission status in the medium-sized city of Tanga, Tanzania, following seven rounds of MDA. The study revealed compelling evidence of a significant reduction in infection and transmission, as indicated by microfilaria (mf) and circulating filarial antigen prevalences that remained notably above the thresholds for discontinuing MDA. This observation paralleled the findings of Mwakitalu et al. [45], who noted a substantial decrease in the LF burden in the metropolitan area of Dar es Salaam, Tanzania. The authors suggested that the reduction could be attributed to a combination of factors, including urban malaria control measures targeting Anopheles vectors, and the relatively short lifespan of the abundant *Cx. quinquefasciatus* vectors in urban settings, widespread use of bed nets and other mosquito-repelling interventions, as well as the impact of mass drug administration. Meyrowitsch et al. [53] discovered that between the two examination points, individuals with low MBL expression genotype had about ten times the frequency of new infections as those with high MBL expression genotype. The study examined the relationship between *Wuchereria bancrofti* infection and Mannose-Binding Lectin (MBL) polymorphisms in the Kwale and Tawalani communities in Northeastern Tanzania. The host's MBL expression genotype seemed to have a significant impact on *Wuchereria bancrofti* infections.

In 2021, John et al. [55] conducted a quantitative cross-sectional study in Lindi District, Tanzania, to assess community engagement in mass drug administration as well as knowledge, attitudes, and practices on filarial lymphedema management. A total of 954 individuals were interviewed, and the results revealed that 78.5% had participated in ≤ rounds, 60% had inadequate information, and 53.7% had negative attitudes about MDA and lymphedema therapy. The study also found that 74% of individuals used inadequate lymphedema management strategies. The study's main findings revealed a lack of knowledge, a negative attitude, and ineffective lymphedema management procedures in the Lindi district. Another community-based cross-sectional study was carried out in Lindi district, Tanzania, in 2022. The primary objective of the study was to assess the prevalence of filarial lymphedema and investigate the factors associated with its occurrence and management. The research findings indicated that the prevalence of filarial lymphedema was measured at 7.8%, with a notable majority of cases falling within the early stages of lymphedema, accounting for 78.4%. Furthermore, it was observed that a significant proportion of patients, approximately 98.6%, presented with lower limb lymphedema. Notably, 46% of the individuals diagnosed with lymphedema exhibited entry lesions as a notable feature. The study also shed light on the frequency of acute dermatolymphangioadenitis attacks among lymphedema patients, with 60.8% of them reporting having experienced such attacks. In addition, 64.8% of these patients reported experiencing one or two of these attacks within the six months preceding the study [51]. 

Bloch et al. [57] conducted a 22-year follow-up research on lymphatic filariasis in Tanzania to investigate immune responsiveness in relation to long-term infection patterns. The study, which included 71 people aged 34 to 74, found that 85.9% of the participants had the same infection status in 1975 and 1996, indicating a substantial tendency to infection throughout time. The data indicate that specific cellular and antibody responses are more closely connected to present infection status than to previous infection. 

In 2022, over 4,905,803 people required MDA as part of DRC’s MDA, with 4,325,413 million people treated with ivermectin plus albendazole, 90% of endemic regions were covered, and 89% of the individuals were treated as per the program target. The country is currently under post-intervention surveillance [69].

## Conclusion

Finally, this review provides a thorough examination of the prevalence of elephantiasis or lymphatic filariasis (LF) in Southern Africa, which includes Angola, Mozambique, Zimbabwe, the Democratic Republic of the Congo (DRC), Zambia, Malawi, and Tanzania. The findings emphasize the significant impact of LF on the quality of life of those affected, as well as the critical need for effective prevention and elimination strategies. Countries such as South Africa, Botswana, Lesotho, Namibia, and Swaziland are non-endemic regions and do not require preventive chemotherapy. The DRC and Tanzania have successfully administered MDA to the people who required it, however, the countries are under post-intervention surveillance. In Mozambique, LF is a major concern, particularly in the Cabo Delgado region, where the highest prevalence was found. Angola and Zimbabwe's anti-LF efforts have been hampered by a lack of rollout of mass drug administration. Hence, LF remains a public health concern, in these countries. Over the years, Zambia has made significant progress in reducing LF cases through mapping surveys and MDA programs, especially in high-prevalence areas. However, due to the non-rollout of MDA, LF incidence in the country has increased. All the people in Malawi have received MDA hence the county is currently under post-intervention surveillance. Proper monitoring is essential to ensure that LF is completely eradicated from Malawi. 

Overall, this review sheds light on the serious challenges posed by LF in Southern Africa and emphasizes the critical importance of sustained efforts, including innovative strategies and ongoing research, to effectively combat and ultimately eliminate this debilitating disease. We can minimize the burden of elephantiasis and improve the well-being of affected persons throughout the area by addressing the unique requirements and conditions of each country.

### Recommendations

- *Strengthen Surveillance and Research Efforts:* Given the limited data in certain SADC countries, it is important to prioritize and invest in robust surveillance and research initiatives. This will provide a more accurate understanding of LF prevalence and distribution, enabling targeted interventions.
- *Enhance Access to Treatment and Care:* Efforts should be made to improve access to specialized care and treatment for individuals affected by LF-related complications like lymphedema and hydrocoele. This includes ensuring the availability of necessary medical supplies and trained healthcare professionals.
- *Community Engagement and Education:* Raising awareness and understanding of LF among local communities is essential. This can be achieved through community-based education programs, involving local leaders, and leveraging existing healthcare infrastructure.
- *Multi-Sectoral Approach:* Addressing LF requires a coordinated effort across various sectors including health, education, sanitation, and environment. Collaborative approaches involving governments, non-governmental organizations, and international partners are important for success.
- *Sustained Mass Drug Administration (MDA)*: Continued implementation of MDA programs, along with rigorous monitoring and evaluation, remains a cornerstone in the fight against LF. Efforts should be made to ensure the coverage and compliance of MDA in all endemic areas.
- *Tailored Interventions:* Recognizing the variability of LF occurrence and impact within countries, interventions should be targeted to specific regions and people. This could include targeted MDA, specialist care for people with advanced problems, and environmental control in high-risk locations.
- *Integration with Other Health Programs:* Integrating LF control efforts with existing health programs, such as those addressing other neglected tropical diseases, can lead to more efficient use of resources and improved healthcare delivery.
- *Monitoring and Evaluation:* Establishing robust monitoring and evaluation frameworks will allow for the tracking of progress, identification of challenges, and adjustment of strategies as needed.

While progress has been made in the fight against LF, particularly through initiatives like GPELF and MDA programs, the burden of this disease remains substantial, particularly in the SADC region. By implementing the above recommendations, stakeholders can work towards a future where LF no longer poses a significant threat to the health and well-being of individuals in Southern Africa.

### Abbreviations

AJO: African Journal Online; aOR: adjusted odds ratio; CFA: circulating filarial antigens; DEC: diethylcarbamazine citrate; DRC: Democratic Republic of the Congo; GPELF: Global Program to Eliminate Lymphatic Filariasis; HIV: human immunodeficiency virus; ICT: immunochromatographic test; KAP: knowledge, attitudes, and practices; LAOSA: Lymphoedema Association of South Africa; LF: lymphatic filariasis; LLINs: long-lasting insecticidal nets; MDA: mass drug administration; mf: microfilaria; MISM: multiple intervention score map; MOM: micro-stratification overlap mapping; NLFEP: National Lymphatic Filariasis Elimination Program; NTDs: neglected tropical diseases; pre-TAS: pre-transmission assessment survey; RAPLOA: rapid assessment method for loiasis; REMO: rapid epidemiological mapping of onchocerciasis; SADC: Southern African Development Community; WHA: World Health Assembly's; WHO: World Health Organization.

## Data Availability

All data generated or analyzed during this study are included in this article
